# Broad adsorption of sepsis-related PAMP and DAMP molecules, mycotoxins, and cytokines from whole blood using CytoSorb^®^ sorbent porous polymer beads

**DOI:** 10.1371/journal.pone.0191676

**Published:** 2018-01-25

**Authors:** Maryann C. Gruda, Karl-Gustav Ruggeberg, Pamela O’Sullivan, Tamaz Guliashvili, Andrew R. Scheirer, Thomas D. Golobish, Vincent J. Capponi, Phillip P. Chan

**Affiliations:** CytoSorbents Corporation, Monmouth Junction, New Jersey, United States of America; Medizinische Universitat Graz, AUSTRIA

## Abstract

**Objective:**

Sepsis is defined as life-threatening organ dysfunction caused by a dysregulated host response to infection. In sepsis and septic shock, pathogen-associated molecular pattern molecules (PAMPS), such as bacterial exotoxins, cause direct cellular damage and/or trigger an immune response in the host often leading to excessive cytokine production, a maladaptive systemic inflammatory response syndrome response (SIRS), and tissue damage that releases DAMPs, such as activated complement and HMGB-1, into the bloodstream causing further organ injury. Cytokine reduction using extracorporeal blood filtration has been correlated with improvement in survival and clinical outcomes in experimental studies and clinical reports, but the ability of this technology to reduce a broader range of inflammatory mediators has not been well-described. This study quantifies the size-selective adsorption of a wide range of sepsis-related inflammatory bacterial and fungal PAMPs, DAMPs and cytokines, in a single compartment, *in vitro* whole blood recirculation system.

**Measurements and main results:**

Purified proteins were added to whole blood at clinically relevant concentrations and recirculated through a device filled with CytoSorb^®^ hemoadsorbent polymer beads (CytoSorbents Corporation, USA) or control (no bead) device *in vitro*. Except for the TNF-α trimer, hemoadsorption through porous polymer bead devices reduced the levels of a broad spectrum of cytokines, DAMPS, PAMPS and mycotoxins by more than 50 percent.

**Conclusions:**

This study demonstrates that CytoSorb^®^ hemoadsorbent polymer beads efficiently remove a broad spectrum of toxic PAMPS and DAMPS from blood providing an additional means of reducing the uncontrolled inflammatory cascade that contributes to a maladaptive SIRS response, organ dysfunction and death in patients with a broad range of life-threatening inflammatory conditions such as sepsis, toxic shock syndrome, necrotizing fasciitis, and other severe inflammatory conditions.

## Introduction

Sepsis has been defined as life-threatening organ dysfunction caused by a dysregulated host response to infection [1]. Numerous factors contribute to this dysregulation, including pro-inflammatory and anti-inflammatory cytokines, pathogen-associated molecular patterns (PAMPs) such as bacterial exotoxins and endotoxins, mycotoxins, damage-associated molecular patterns (DAMPs) released by injured cells, and host-specific factors such as activated complement and procalcitonin. Complex interactions involving such substances can lead to severe immune system dysfunction ranging from a destructive maladaptive systemic inflammatory response syndrome (SIRS) to advanced immunosuppression.

PAMPs often trigger the initial sepsis cascade, evoking both innate and cell-mediated immune responses. Inflammation from upregulation of pro-inflammatory cytokines, activated complement, and other mediators, coupled with direct PAMP-induced injury to cells, can release DAMPs. This broad class of endogenous molecules is normally found only intracellularly. Once released into the bloodstream [2], however, DAMPs, like PAMPs, trigger immune responses through pattern recognition receptors [3]. Persistent circulating DAMP elevations contribute to organ injury [4] and identify those patients at highest risk of multiple organ dysfunction syndrome and death in community-acquired pneumonia and sepsis [5].

The foregoing observations suggest that removing not only cytokines, but PAMPs, DAMPs, and other inflammatory mediators from blood may modulate systemic inflammation during sepsis, prevent or treat organ failure, and improve patient outcomes. A sorbent technology developed to effect such removal is CytoSorb^®^ (CS; CytoSorbents Corporation, Monmouth Jct., NJ), an extracorporeal cytokine adsorber composed of highly porous poly(styrene-*co*-divinylbenzene) hemoadsorbent beads. Via a combination of size exclusion and hydrophobic interactions, the device adsorbs proteins and other substances within a 10–60 kDa molecular weight range leading to the hypothesis that the device could also adsorb PAMPs, DAMPs, and toxins [6, 7]. While case reports have described positive outcomes with CytoSorb use as a cytokine adsorber in septic shock [8–17], toxic shock [18], necrotizing fasciitis [10], cardiac surgery [19, 20], and liver failure [21, 22], investigation into removal of inflammatory mediators other than cytokines has been limited. Thus, this study was performed to quantify the ability of CS polymer beads to adsorb a broad selection of PAMPs, DAMPs and mycotoxins, as well as cytokines, from whole blood in a single-compartment, *in vitro* recirculation system.

## Materials and methods

### Studied analytes

These experiments examined the hemoadsorption profiles of four cytokines, four DAMPs, three PAMPs, and two mycotoxins. These factors were selected for evaluation because they are frequently present at high levels, and have been associated with detrimental effects, in septic patients.

The studied DAMPs included high mobility group box-1 (HMGB-1) [23], procalcitonin [24], S100 protein [25], and C5a [26]. Extracellular HMGB-1 is an indicator of tissue necrosis and has been associated with increased risk of sepsis and multi-organ dysfunction syndrome after blunt chest trauma [23]. Procalcitonin is involved in tissue transmigration and monocyte activation [24]. S100A8 and S100A9 homodimers and heterodimers bind to and signal directly via the toll-like receptor 4/lipopolysaccharide receptor complex, with S100A8 as the active component [25]. The complement system forms part of the host defense against pathogens, but excessive levels of C5a and other activated factors can cause tissue injury and adverse outcomes in sepsis [26].

The PAMPs studied comprised *Staphylococcus* α-toxin (α-hemolysin), toxic shock syndrome toxin-1 (TSST-1), and Streptococcus pyrogenic exotoxin B (SpeB). Such bacterial exotoxins are potent mediators of both direct and indirect injury to the host, but their role is often overlooked. α-hemolysin, a key virulence factor produced by most *Staphylococcus aureus* isolates, forms pores in cell membranes and causes rapid tissue destruction [27]. TSST-1, a superantigen, non-specifically activates T-cells, resulting in hyperactivation of the immune response and excessive cytokine release [28]. SpeB, a virulence factor synthesized by *Streptococcus pyogenes*, inhibits phagocytic activity by cleaving and degrading host immunoglobulin and activated complement components [29].

The studied mycotoxins, aflatoxin and trichothecene T-2 toxin, are typical low-molecular weight (<1kDa) products of disseminated fungal infections that often arise at sites of previous or concomitant bacterial infection. These substances can suppress humoral and cellular immunity, trigger cell death and apoptosis, and promote tissue breakdown, resulting in organ failure, e.g., acute hepatic failure [30, 31].

### *In vitro* recirculation system and quantification of protein removal

The solution-depletion method [32] was used to quantify protein removal in this study because it enables a well-controlled simulation of the sorbent’s potential ability to remove factors from the vascular compartment without the complexity of refill from extravascular spaces or *de novo* synthesis, helping to establish a baseline reference for animal or clinical studies. In brief, purified inflammatory proteins were added to 3.8% citrated whole bovine blood (Lampire Biological Laboratories, Pipersville, PA; AAALAC accreditation #001032, USDA #23-R-0122, 23-B-0020 and NIH Office of Lab Animal Welfare OLAW #A3997-01) at typical clinical concentrations. The proteins were recirculated with a peristaltic pump through a CS polymer-filled device or control (no bead) device for five hours. The blood volume to polymer ratio was maintained at 13:3 for all size devices to reflect the clinical treatment of an adult patient with the 300 mL device. Typical flow rates for the 300 mL device in clinical use range from 150–500 mL/min, meaning that blood turnover can range from every 8 minutes to every 26 minutes. Purified recombinant cytokines (Sigma-Aldrich, St. Louis, MO): MIP1-α at 400 pg/mL, IL-6 at 3000 pg/mL, IFN-γ at 400 pg/mL, and TNF-α at 800 pg/mL, were added together into 927 mL of bovine blood and recirculated through a 70 mL polymer-filled device or control device at 140 mL/min for five hours. Due to more limited availability of materials, purified recombinant DAMP and PAMP proteins were individually spiked into 265 mL of bovine blood and recirculated through a 20 mL device at a flow rate of 40 mL/min. Initial concentrations were: HMGB-1 (R&D Systems, Minneapolis, MN) at 100 ng/mL [23], S100A8 (ACROBiosystems, Newark, DE) at 50 ng/mL [25], complement C5a (R&D Systems) at 25 ng/mL [26], procalcitonin (Sigma-Aldrich) at 16 ng/mL [24], TSST-1 (Toxin Technology, Inc., Sarasota, FL) at 2 μg/mL [33], SpeB (Toxin Technology) at 100 ng/mL [28]. A 10 mL device at a flow rate of 20 mL/min in a blood volume of 133 mL was used for the mycotoxin recirculation experiments. The initial concentrations of *Aspergillus flavus* aflatoxin B1 (Sigma-Aldrich) was 10 μg/mL [34] and T-2 toxin (Enzo Life Sciences, Farmingdale, NY) were 10 μg/mL [35]. Plasma was prepared and ELISA was performed following manufacturer instructions (cytokines, S100, and C5a, duosets [R&D Systems]; procalcitonin [Sigma-Aldrich], HMGB-1 [Chondrex, Inc., Redmond, WA]; bacterial toxins (Toxin Technology); and aflatoxin (Helica BiosystemsInc., Santa Ana, CA). Due to the presence of anti-hemolysin antibodies in blood obtained from adult bovine donors, fetal bovine serum (90 mL) was used for the α-hemolysin (Toxin Technology) recirculation experiment (1.5μg/mL with a 10 mL polymer device). α-hemolysin was quantitated by ELISA (Neogen Corp., Lansing, MI). Further details of the protocols can be found at: http://dx.doi.org/10.17504/protocols.io.kurcwv6.

### Statistics

Data are described as mean ± standard error of the mean (SEM) for the percent changes versus baseline values (t = 0). Differences among groups were compared using two-way ANOVA. Statistical significance of individual time points between control and CS devices was determined by multiple t-tests using the Holm-Sidak method with alpha = 5.000%. Each time point was individually analyzed without assuming a consistent standard deviation. Significant differences were regarded as p<0.05. All statistical analyses were performed with the aid of GraphPad PRISM^®^.

## Results

**Fig 1:**
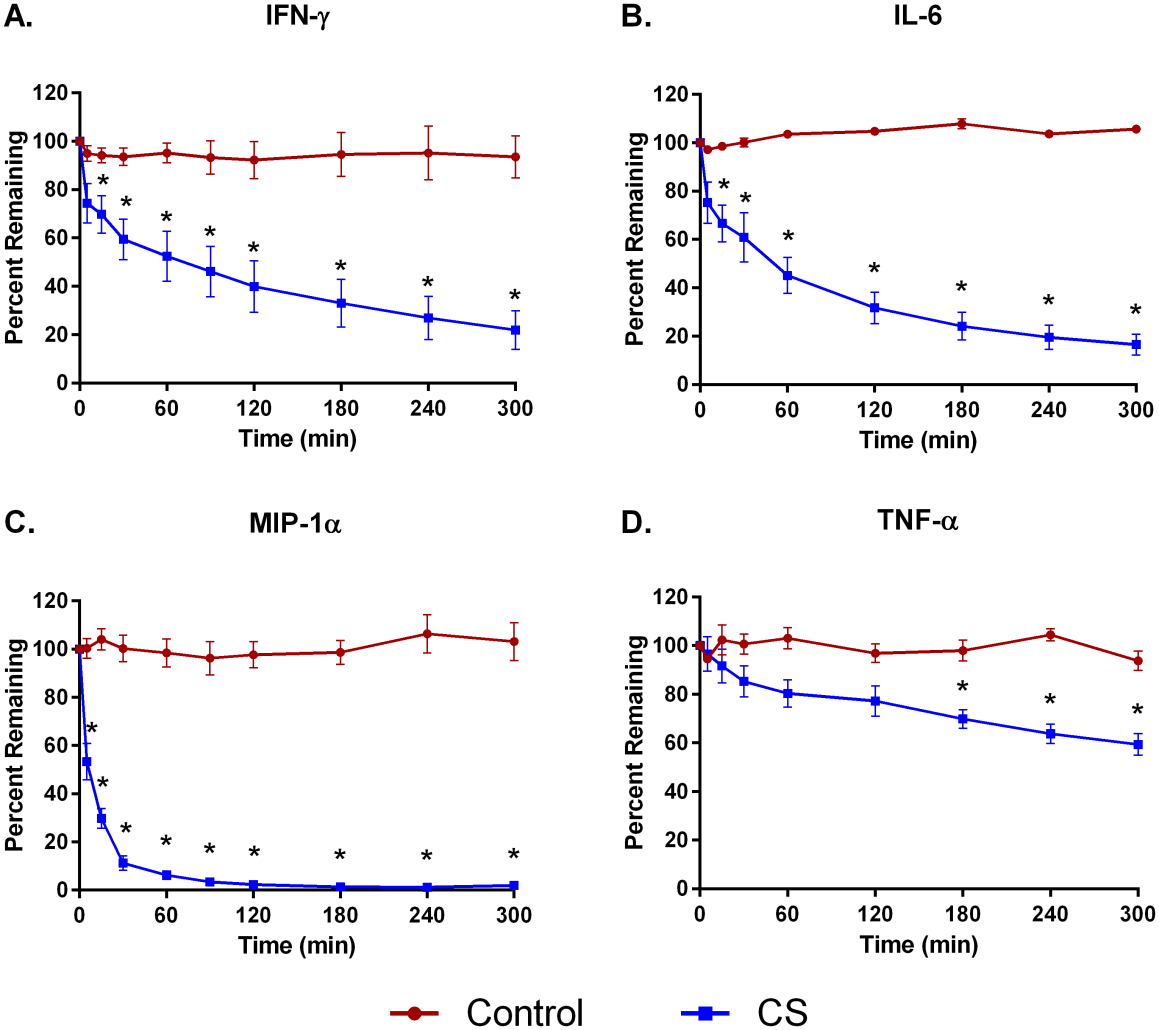
Adsorption of cytokines from whole blood with CS hemoadsorptive polymer beads or a control (no polymer) device. Percent remaining from the mean ± SEM of 4 runs. * p<0.05.

**Fig 2:**
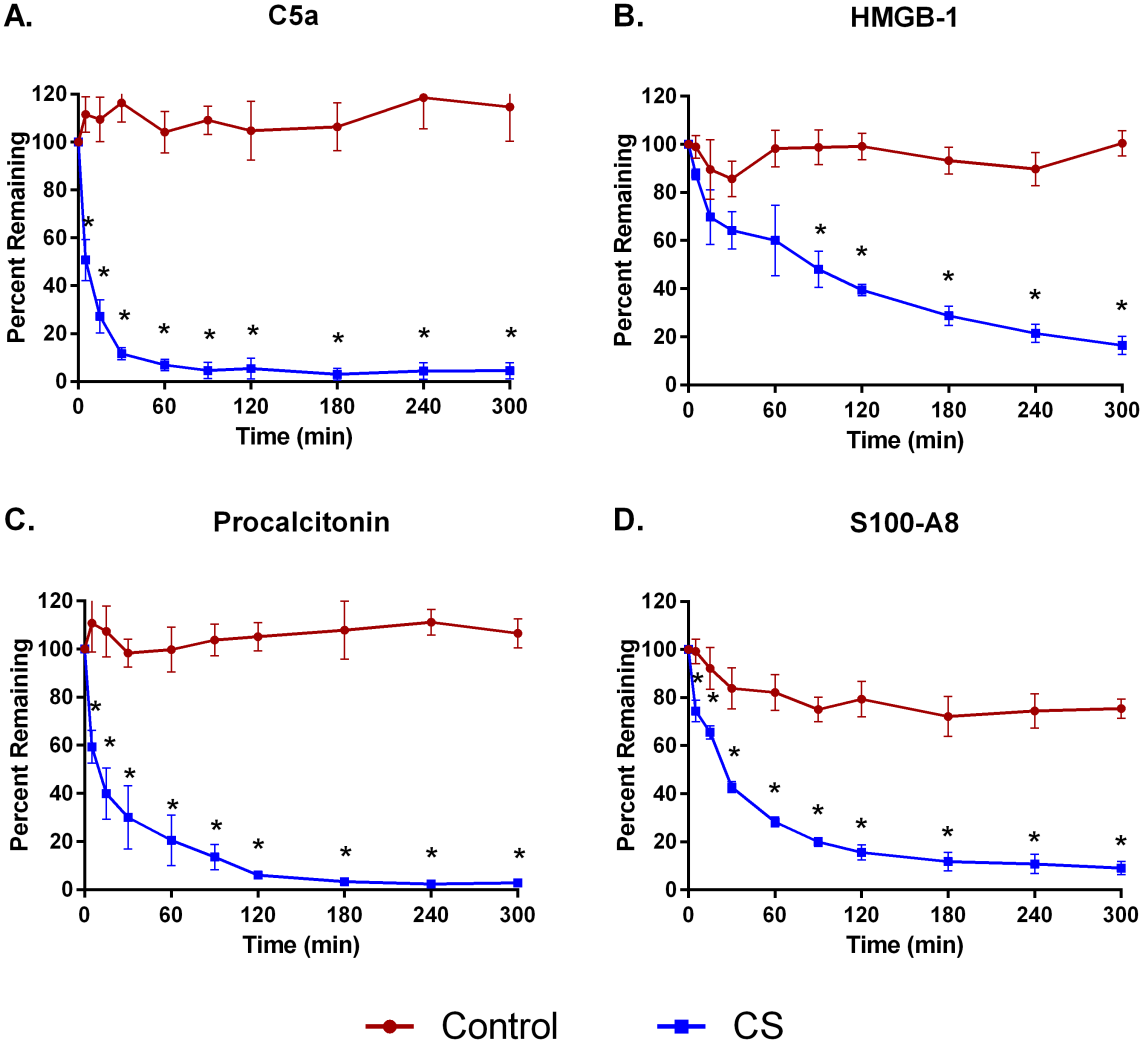
Adsorption of DAMPs from whole blood with CS hemoadsorptive polymer beads or a control (no polymer) device. Percent remaining from the mean ± SEM of 4 runs per analyte. * p<0.05.

**Fig 3:**
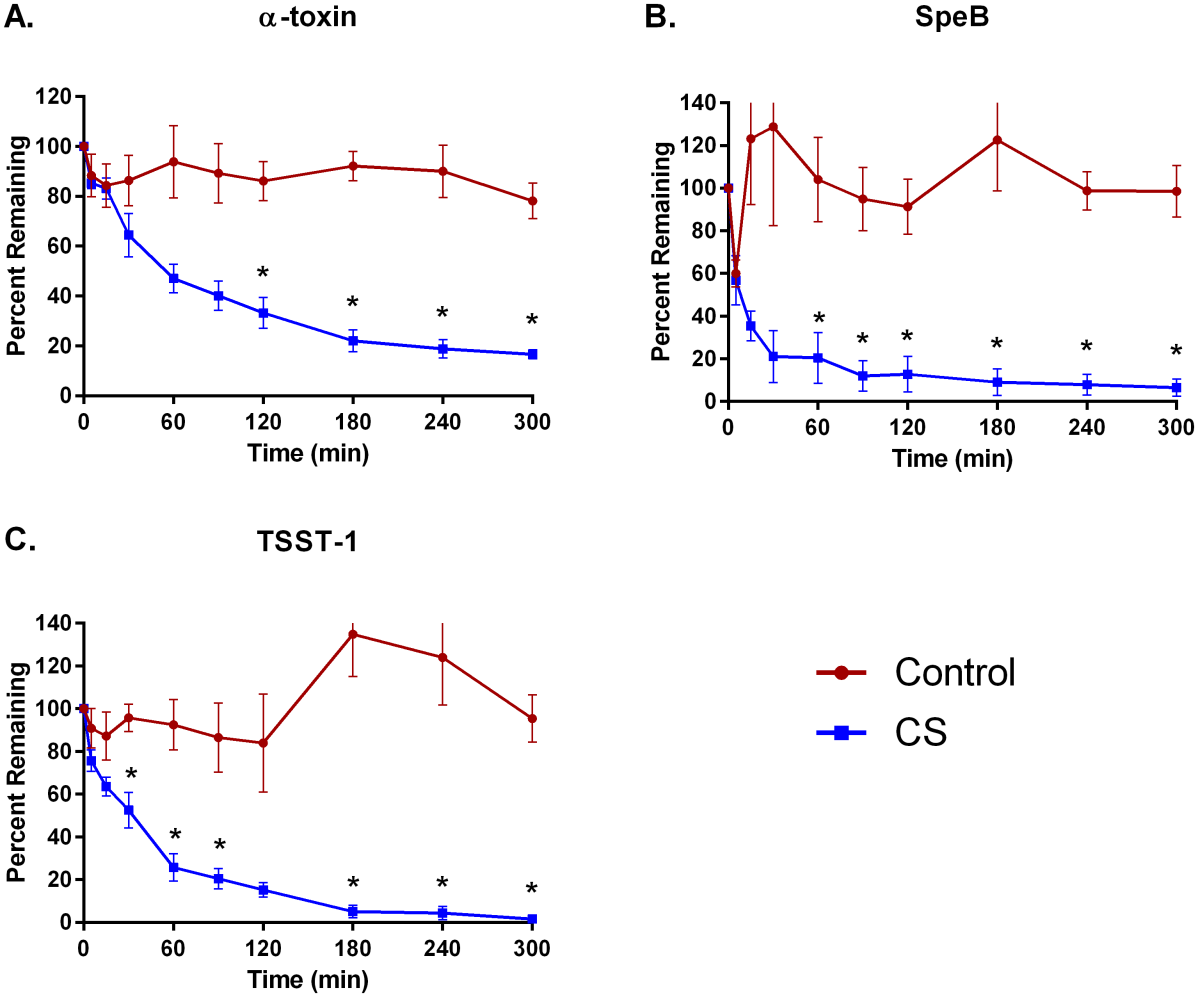
Adsorption of bacterial PAMPs with CS hemoadsorptive polymer beads or a control (no polymer) device from whole blood spiked with *S*. pyogenic exotoxin B, *Staph* TSST-1 or serum with *Staph aureus* alpha-toxin. Percent remaining from the mean ± SEM of 4 runs. * p<0.05.

**Fig 4:**
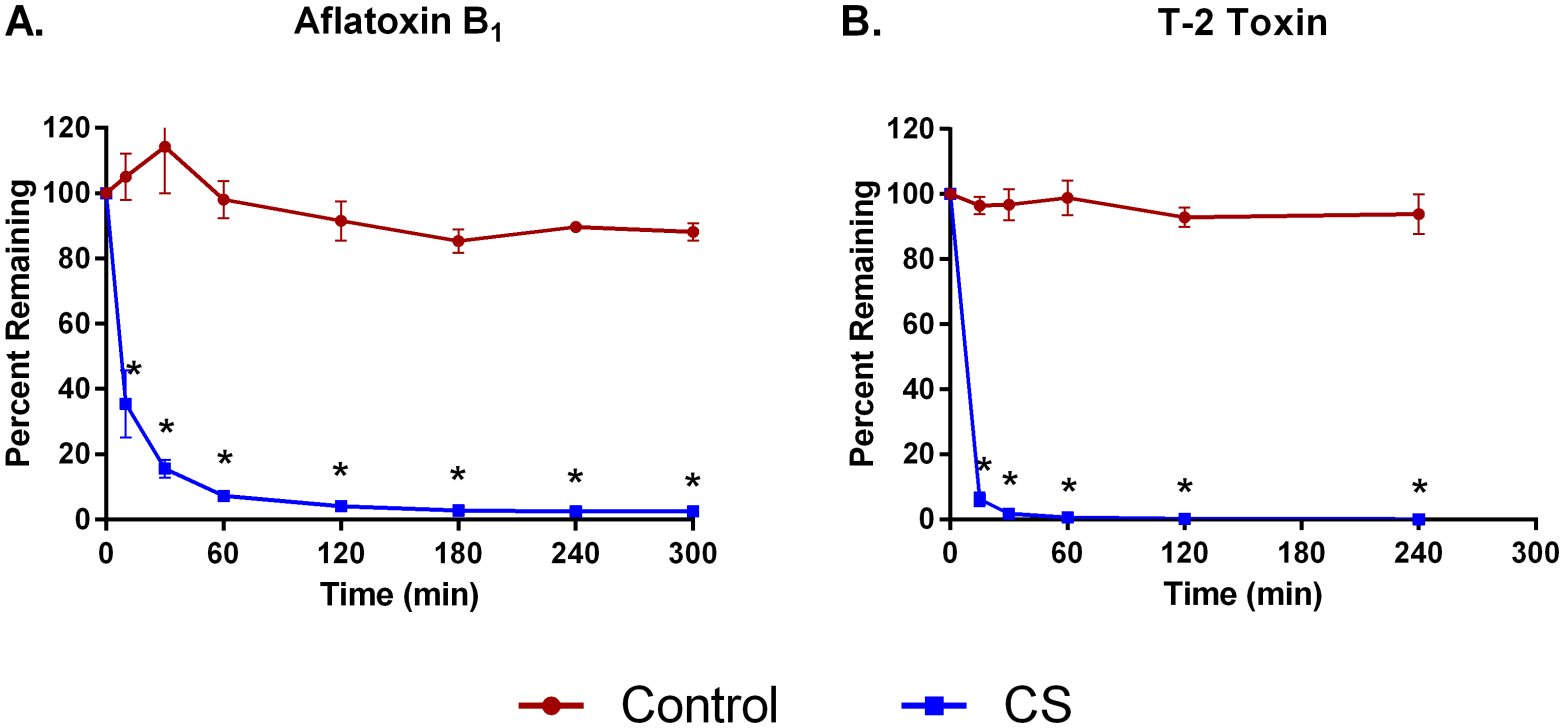
Adsorption of mycotoxins with CS hemoadsorptive polymer beads or a control (no polymer) device from whole blood spiked with *A*. *flavus* aflatoxin B1 (10 mg/L) or *Fusarium* T-2 toxin (10 mg/L). Percent remaining from the mean ± SEM of 4 runs. * p<0.05.

Hemoperfusion of whole blood through CS polymer bead devices for five hours reduced the levels of cytokines MIP1-α, IL-6, and IFN-γ by 98±4.0%, 91±3.0%, and 82±15%, respectively (Fig 1). TNF-α removal was not as efficient, reaching only 41±4.4% by 5 hours. The CS device removed 83%-98% of DAMPS C5a, HMGB-1, procalcitonin, and S100-A8 (Fig 2) and bacterial PAMPS α-toxin, SpeB, and TSST-1 (Fig 3). Removal of aflatoxin and T-2 toxin was particularly efficient, achieving levels of over 95% and 99%, respectively, within 2 hours (Fig 4). In contrast, levels of all the inflammatory mediators tested remained fairly constant in recirculation experiments with a control device (Figs 1–4).

Removal efficiency correlated with the size of the analyte (S1 Fig). The CS polymer device removed over 90% of the small toxins, such as aflatoxin (0.3 kDa) and T-2 toxin (0.5 kDa), and proteins 20 kDa or less, MIP1-α (8 kDa), C5a (8.2 kDa), procalcitonin (13 kDa), and S100A8 (20 kDa) The adsorption of proteins between 20 and 50 kDa, such as IL-6 (26 kDa), IFN-γ (25 kDa), HMGB-1 (25 kDa), TSST-1 (24 kDa), α-toxin (33 kDa) and SPE B (40 kDa), was more variable, demonstrating 79%-97% removal, with 41% of the 52 kDa TNF-α adsorbed (S1 File).

## Discussion

Sepsis is a highly heterogeneous disease with severity and progression of illness dependent upon a myriad of interacting factors. These factors [36] include, for example, the microbial insult, which may be of bacterial (gram-positive and gram-negative), viral, or fungal origin; the pathogen load; expression of virulence factors; host factors such as age, genetic characteristics, and comorbidities; the site of infection; and the elapsed time since the initial infection. This complexity creates a highly dynamic and unstable situation that has so far confounded therapeutic efforts targeted against single factors. This complexity is also what has driven interest in broad-spectrum blood purification strategies to treat sepsis by reducing a wide range of PAMPs and DAMPs, correcting the imbalance of pro-inflammatory and anti-inflammatory factors, and re-establishing immune homeostasis. When efficacious, and used with antibiotics, broad-spectrum blood purification therapies could lead to a more comprehensive and improved approach to sepsis.

Recognition of the multitude of damaging roles played by these soluble mediators has over the past few decades led to the evolving application of broad-spectrum extracorporeal blood purification techniques to remove excessive inflammatory mediators and toxins in sepsis and related conditions [36–38]. However, the effectiveness of such modalities generally has been poor and constrained by the technologies used. For example, standard hemodialysis, hemodiafiltration, and hemofiltration have demonstrated only limited removal of a broad range of cytokines and inflammatory toxins [38, 39]. Initial enthusiasm for high-volume hemofiltration (HVHF) for this purpose has dissipated due to the significant costs associated with the required large volumes of ultrapure replacement solution, the risk of severe electrolyte disturbances, and the lack of hemodynamic or survival benefits [40]. Although high-molecular weight cut-off filters have shown some promise in sepsis and related disorders [41, 42], numerous questions remain unanswered regarding the timing, duration, and frequency of administration and the optimal membrane characteristics (molecular weight cut-off, surface area, composition). The “High Cut-Off Continuous Venovenous Hemodialysis In Septic Patients Treated For Acute Renal Failure After Systemic Inflammatory Response Syndrome/Septic Shock” study [43] was terminated early, after an interim analysis showed no difference in 28-day mortality, vasopressor use, duration of mechanical ventilation, or intensive care unit length-of-stay in hemodialysed patients versus controls.

Unlike membrane-based technologies that work by diffusion or convection, hemoadsorptive devices are filled with sorbent materials that have much greater surface area. These materials can adsorb substances from blood based on affinity, ionic interaction, polarity, or other surface adsorptive mechanisms. A hemoadsorptive device containing polyester fibers bound to polymyxin B has been applied to remove lipopolysaccharide endotoxin found in gram-negative infections [44, 45]. Mixed results have been seen in clinical studies, possibly because of the small window for intervention before immune response activation.

Using another hemoadsorptive technology, CS porous polymer beads, this report demonstrates in an *in vitro* system the reduction of a broad range of toxic PAMPs, DAMPs and mycotoxins from whole blood. Such reduction relies on a combination of pore capture and surface adsorption, without use of biological ligands. The defined porous structure of the CS beads contributes to selectivity by limiting adsorption of substances greater than approximately 65 kDa, such as albumin [46] and immunoglobulins (>150 kDa) [20]. Proteins in the approximate range of 10–60 kDa fit inside the pores where they adsorb onto the internal polymer surface. Surface adsorption by the hydrophobic CS polymer, through a combination of non-polar interactions, hydrogen bonding, and van der Waals forces [32], further discriminates in favor of removing hydrophobic substances over hydrophilic ones. This mechanism is primarily what governs CS adsorption of detrimental small molecules such as mycotoxins, antibiotics, or β2-microglobulin [47, 48] as well as beneficial molecules such as Vitamin B12 [S2 Fig]. Finally, *in vitro* investigations indicate that the degree of adsorption of a given protein in a mixed solution of proteins relates linearly to that protein’s relative concentration in the solution, i.e., there is greater adsorption at high concentrations, and less adsorption at low concentrations [49, 50]. This observation suggests that in the blood of patients with sepsis, the low concentrations of non-target substances relative to the elevated levels of target substances, potentially might limit the removal of the former by the adsorbent CS beads. Such a mechanism might serve to minimize the risk of overtreatment.

The ability to remove a broad range of PAMPs and DAMPs supports several presumed mechanisms by which blood purification with the CytoSorb^®^ adsorber may help to stabilize critically-ill sepsis patients. A first, direct mechanism would be reduction in levels of mycotoxins, bacterial exotoxins, and other PAMPs that cause direct tissue injury and/or lead to hyperactivation of the immune response and SIRS. Secondly, such a reduction in inflammatory mediators could lead to diminished levels of pro-inflammatory cytokines that can contribute to direct tissue injury and organ dysfunction, e.g., cell apoptosis, endothelial injury, capillary leak syndrome, and SIRS [2, 3]. Thirdly, the resultant decrease in the “noise” of circulating cytokines could “boost the signal” of cytokines in infected tissue. Thereby, activated leukocytes would be re-directed to the actual site of infection, improving source control while decreasing the risk of leukocyte-mediated injury to non-infected organs [51, 52]. Lastly, in settings of bacterial/fungal co-infection such as invasive pulmonary disease [31], PAMP removal might diminish, or avoid induction of, mycotoxin production and any resultant increase in fungal virulence [31]. A combination of these mechanisms may underlie the amelioration of disease by the adsorber in patients with sepsis and related conditions, e.g., toxic shock syndrome and necrotizing fasciitis [4–6, 12–15].

We acknowledge that these experiments have significant limitations, including being performed in an *in vitro* hemoperfusion system with non-septic blood, without a natural refill rate to challenge the CS device. A randomized controlled study [46] of CytoSorb use in mechanically-ventilated septic patients found no difference in systemic levels of IL-6 between patients receiving treatment with the hemoadsorption device as compared to standard of care, despite demonstration of median IL-6 elimination rates of 5%-18% per pass of blood through the device. This observation is thought to be due to cytokines shifting from the interstitium into the bloodstream as described by the cytokinetic theory [38, 52, 53]. Another limitation of the *in vitro* nature of our study was an inability to correlate PAMP and DAMP removal with anti-inflammatory effect or other clinical benefit, or to answer clinical questions such as optimal timing of CS therapy. Further clinical studies are warranted to elucidate these relationships and issues in patients with sepsis and related conditions.

## Supporting information

S1 Fig Correlation of protein size with adsorption with CS polymer at 60 minutes. Protein area (nm^2^) was derived based on molecular weight using the calculator: http://www.calctool.org/CALC/prof/bio/protein_length. Trend line is best linear fit.
(TIF)

S2 Fig Adsorption of Vitamin B12 (300 μg/mL) from bovine whole blood (265 mL) when recirculated through a 20 mL CS hemoadsorptive polymer beads or a control (no polymer) device at 40 mL/min. Percent remaining from the mean ± SEM of 4 runs. * p<0.05.
(TIF)

S1 File Supporting data. (PDF)
