# Supplementary figures and images for: Broad adsorption of sepsis-related PAMP and DAMP molecules, mycotoxins, and cytokines from whole blood using CytoSorb® sorbent porous polymer beads
Source: PLoS One. 2018 Jan 25;13(1):e0191676. doi: 10.1371/journal.pone.0191676 (PMC5784931; doi:10.1371/journal.pone.0191676)

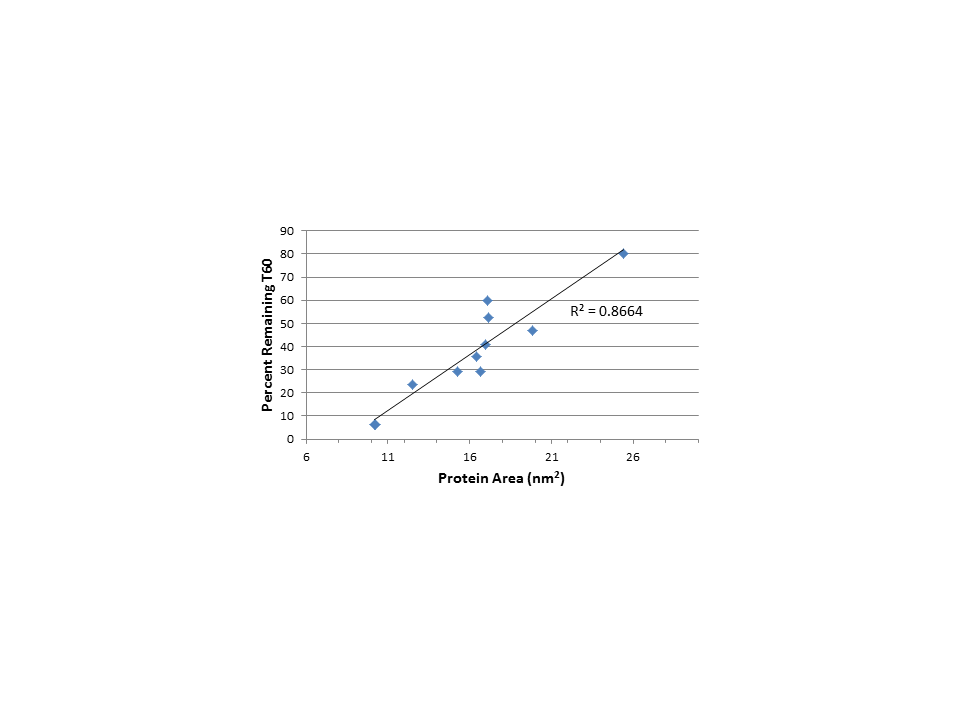

Supplement: S1 Fig — Protein area (nm2) was derived based on molecular weight using the calculator: http://www.calctool.org/CALC/prof/bio/protein_length. Trend line is best linear fit. (TIF) [file pone.0191676.s001.tif]
