# Supplementary material for: Broad adsorption of sepsis-related PAMP and DAMP molecules, mycotoxins, and cytokines from whole blood using CytoSorb® sorbent porous polymer beads
Source: PLoS One. 2018 Jan 25;13(1):e0191676. doi: 10.1371/journal.pone.0191676 (PMC5784931; doi:10.1371/journal.pone.0191676)
Supplement: S1 File — (PDF) [file pone.0191676.s003.pdf]

Supplementary Data

Vitamin B12	Concentration (µg/mL)						Concentration (µg/mL)					
Time (min)	Control 1	Control 2	Control 3	Control 4	Average Control	SD Control	CytoSorb 1	CytoSorb 2	CytoSorb 3	CytoSorb 4	Average CytoSorb	SD CytoSorb
0	129.0	119.0	85.0	85.0	104.5	22.9	456.3	372.7	107.0	449.6	346.4	164.0
5	130.0	124.0	85.5	88.0	106.9	23.4	134.5	57.0	33.0	89.7	78.5	43.9
15	127.0	121.5	87.5	83.0	104.8	22.7	76.4	54.6	19.0	98.4	62.1	33.8
30	125.5	115.5	84.0	83.0	102.0	21.8	35.3	31.9	10.0	79.1	39.1	28.9
60	129.0	119.0	91.5	86.0	106.4	20.9	16.8	5.8	3.0	48.5	18.5	20.9
90	132.0	128.0	92.5	93.0	111.4	21.6	11.1	0.0	7.5	37.2	13.9	16.2
120	135.5	111.5	95.5	93.0	108.9	19.6	17.6	0.0	7.5	25.8	12.7	11.3
180	136.0	120.0	97.5	98.0	112.9	18.6	25.7	0.0	11.5	14.5	12.9	10.6

Aflatoxin B1	Concentration (ng/mL)						Concentration (ng/mL)					
Time (min)	Control 1	Control 2	Control 3	Control 4	Average Control	SD Control	CytoSorb 1	CytoSorb 2	CytoSorb 3	CytoSorb 4	Average CytoSorb	SD CytoSorb
0	7617	7901	16000	16200	11930	4818	14629	18079	15400	15300	15852	1524
10	7026	9423	18450	15100	12500	5215	3584	2460	6700	9200	5486	3057
30	11896	7957	15100	17100	13013	3995	1898	1619	3120	3040	2419	772
60	6230	7804	16700	17400	12034	5835	624	741	1640	1510	1129	520
120	5866	8089	13800	16300	11014	4857	495	311	810	870	622	264
180	11900	7558	12800	13000	11315	2550	171	218	600	730	430	277
240	11900	7152	14700	14000	11938	3405	189	170	602.5	570	383	235
300	11900	7449	13000	14400	11687	3005	182	149	577.5	627.5	384	253

T2 Toxin	Concentration (ppb)						Concentration (ppb)					
Time (min)	Control 1	Control 2	Control 3	Control 4	Average Control	SD Control	CytoSorb 1	CytoSorb 2	CytoSorb 3	CytoSorb 4	Average CytoSorb	SD CytoSorb
0	115827.5	127748.0	116159.5	114597.5	118583.1	6146.7	115827.5	115827.5	123698.5	116159.5	117878.3	3883.3
15	110336.5	116761.0	110391.0	119221.0	114177.4	4516.7	4718.5	1456.5	12174.6	10874.3	7306.0	5077.7
30	99858.5	124360.0	109288.5	125283.0	114697.5	12313.5	23.5	877.0	4985.9	2666.2	2138.1	2194.7
60	96621.0	129143.0	125026.5	118267.0	117264.4	14474.2	0.0	0.0	1666.0	841.0	626.7	798.2

120	102840.0	112076.0	109288.5	115493.5	109924.5	5361.5	0.0	0.0	601.0	284.6	221.4	286.4
240	89516.5	123042.5	110391.0	122177.5	111281.9	15615.7	0.0	0.0	171.6	2.3	43.5	85.4

IL-6	Concentration (pg/mL)						Concentration (pg/mL)					
Time (min)	Control 1	Control 2	Control 3	Control 4	Average Control	SD Control	CytoSorb 1	CytoSorb 2	CytoSorb 3	CytoSorb 4	Average CytoSorb	SD CytoSorb
0	2030	2030	2026	2632	2179	302	2659	3898	2273	4376	3302	997
5	2113	1893	2018	2562	2147	291	1979	2501	1950	1872	2076	287
15	2152	2043	2014	2338	2137	147	1641	2255	1604	1600	1775	321
30	2195	2055	1968	2426	2161	200	1334	2002	1400	1159	1474	367
60	2202	2072	2086	2290	2163	103	1034	1426	1099	826	1096	249
120	2314	2164	2109	2285	2218	97	658	821	680	463	655	147
180	2237	2233	2270	2407	2287	82	491	542	469	324	457	93
240	2403	2200	2111	2557	2318	201	331	426	361	252	342	72
300	2365	2435	2138	2489	2357	154	299	343	280	213	284	54

MIP-1α	Concentration (ng/mL)						Concentration (ng/mL)					
Time (min)	Control 1	Control 2	Control 3	Control 4	Average Control	SD Control	CytoSorb 1	CytoSorb 2	CytoSorb 3	CytoSorb 4	Average CytoSorb	SD CytoSorb
0	256	186	240.0	259.9	235.5	34.1	216.9	130	357	137	210.2	105.5
5	249	208	223.0	217.9	224.5	17.5	142.4	79	230	96	136.9	67.6
15	281	209	213.0	200.6	225.9	37.1	79.5	52	132	92	88.9	33.3
30	238	217	220.0	191.9	216.7	19.0	34.3	25	70	96	56.3	32.8
60	230	215	210.0	213.3	217.1	8.9	9.9	15	22	53	25.0	19.3
120	230	208	201.0	210.9	212.5	12.4	0.0	10	0	43	13.3	20.4
180	251	203	214.0	200.1	217.0	23.4	0.0	9	0	30	9.8	14.2
240	279	213	214.0	201.2	226.8	35.3	0.0	8	0	23	7.8	10.8
300	278	214	218.0	196.5	226.6	35.5	0.0	9	0	21	7.5	9.9

IFN-ɣ	Concentration (pg/mL)						Concentration (pg/mL)					
Time (min)	Control 1	Control 2	Control 3	Control 4	Average Control	SD Control	CytoSorb 1	CytoSorb 2	CytoSorb 3	CytoSorb 4	Average CytoSorb	SD CytoSorb
0	68.9	72.1	95.0	101.0	84.2	16.1	104.0	80.2	434.2	104.0	288.1	214.9
5	59.6	66.2	87.0	93.0	76.5	16.1	71.5	73.7	380.0	78.0	237.7	189.0

15	61.9	62.1	89.0	94.0	76.7	17.2	67.0	65.2	366.3	73.0	226.3	181.0
30	58.2	64.4	87.0	89.0	74.6	15.6	56.0	57.1	351.2	56.0	204.3	171.3
60	57.8	63.9	83.0	87.0	72.9	14.2	46.0	46.5	351.7	48.0	197.7	174.1
120	52.0	55.4	83.0	87.0	69.3	18.2	20.5	28.9	307.7	31.0	168.8	165.3
180	53.4	50.4	76.0	86.0	66.4	17.4	14.0	20.2	254.0	21.0	151.5	156.9
240	52.0	43.0	78.0	85.0	64.5	20.2	9.5	11.1	223.6	14.0	129.2	136.9
300	52.0	36.5	80.0	86.0	63.6	23.4	9.5	8.7	193.6	8.0	110.8	118.8

TNF-α	Concentration (pg/mL)						Concentration (pg/mL)					
**Time (min)**	**Control 1**	**Control 2**	**Control 3**	**Control 4**	**Average Control**	**SD Control**	**CytoSorb 1**	**CytoSorb 2**	**CytoSorb 3**	**CytoSorb 4**	**Average**	**SD**
0	176.2	280.0	104.4	389.0	237.4	124.1	200.4	751.0	703.9	545.1	550.1	249.2
5	166.3	269.0	99.4	401.0	233.9	131.4	179.9	701.4	648.4	493.6	505.8	234.5
15	170.1	339.0	96.2	395.0	250.1	140.2	170.3	715.2	603.5	495.1	496.0	235.0
30	174.9	316.0	99.5	407.0	249.3	138.2	159.0	657.2	529.9	447.9	448.5	211.3
60	177.1	325.0	102.5	418.0	255.6	142.3	152.0	646.2	549.8	425.2	443.3	214.3
120	164.6	245.0	105.8	430.0	236.4	141.1	138.5	647.7	479.6	489.1	438.7	214.5
180	156.9	257.0	110.4	421.0	236.3	137.5	128.8	606.7	463.8	363.2	390.6	201.1
240	173.7	304.0	106.8	427.0	252.9	142.1	122.2	557.3	398.5	340.1	354.5	180.0
300	170.3	229.0	102.1	400.0	225.3	127.5	131.1	516.5	339.6	328.3	328.9	157.5

α-toxin	Concentration (ng/mL)						Concentration (ng/mL)					
**Time (min)**	**Control 1**	**Control 2**	**Control 3**	**Control 4**	**Average Control**	**SD Control**	**CytoSorb 1**	**CytoSorb 2**	**CytoSorb 3**	**CytoSorb 4**	**Average CytoSorb**	**SD CytoSorb**
0	727.8	820.6	1055.4	746.2	837.5	150.7	1231.9	1235.8	788.3	860.3	1029.1	238.3
5	773.7	734.3	689.9	689.3	721.8	40.5	1010.7	1133.6	673.0	707.6	881.2	226.6
15	788.3	624.3	713.9	636.0	690.6	76.3	928.7	1174.2	621.9	717.6	860.6	245.2
30	737.3	800.7	598.5	669.6	701.5	87.1	624.9	1110.1	444.5	522.2	675.4	299.0
60	568.4	1062.0	671.5	777.4	769.8	212.6	557.9	788.3	299.9	354.4	500.1	221.9
90	584.7	849.5	632.0	843.7	727.5	138.9	686.3	363.5	259.6	366.6	419.0	185.0
120	659.6	664.6	716.7	784.0	706.2	57.9	579.8	255.0	197.0	348.0	345.0	168.5
180	766.5	708.1	831.9	733.8	760.1	53.5	379.6	119.9	192.8	201.5	223.4	110.3
240	818.4	617.1	732.3	769.0	734.2	85.7	189.2	164.6	233.7	147.5	183.7	37.4

300	657.2	571.2	661.1	672.6	640.5	46.7	189.8	253.1	147.2	105.0	173.8	63.2

S100-A8	**Concentration (ng/mL)**						**Concentration (ng/mL)**					
**Time (min)**	**Control 1**	**Control 2**	**Control 3**	**Control 4**	**Average Control**	**SD Control**	**CytoSorb 1**	**CytoSorb 2**	**CytoSorb 3**	**CytoSorb 4**	**Average CytoSorb**	**SD CytoSorb**
0	74.9	76.9	19.5	32.4	50.9	29.3	17.8	24.3	21.0	10.8	18.5	5.8
5	76.5	71.1	21.9	29.2	49.7	28.1	14.5	19.9	14.8	6.9	14.0	5.4
15	76.2	60.9	21.8	24.6	45.9	27.0	10.9	17.1	14.8	6.5	12.3	4.6
30	73.2	55.7	19.4	21.3	42.4	26.4	7.2	12.1	8.1	4.5	8.0	3.1
60	72.9	68.6	12.2	25.8	44.9	30.4	5.4	8.0	5.2	2.7	5.3	2.2
90	66.5	58.6		22.8	49.3	23.3	3.1	5.5			4.3	1.7
120	73.0	65.7	13.3	21.5	43.4	30.3	2.7	3.9	1.7	2.5	2.7	0.9
180	64.6	66.0	10.2	20.8	40.4	29.1	1.8	2.9	0.7	2.3	1.9	1.0
240	65.3	63.8	10.6	23.7	40.8	27.9	1.3	2.2	0.9	2.4	1.7	0.7
300	63.8	60.3	12.9	23.3	40.1	25.8	1.1	1.6	1.3	1.9	1.5	0.3

C5a	**Concentration (ng/mL)**						**Concentration (ng/mL)**					
**Time (min)**	**Control 1**	**Control 2**	**Control 3**	**Control 4**	**Average Control**	**SD Control**	**CytoSorb 1**	**CytoSorb 2**	**CytoSorb 3**	**CytoSorb 4**	**Average CytoSorb**	**SD CytoSorb**
0	85.4	69.7	15.9	60.7	57.9	29.8	70.0	64.2	51.5	53.0	59.7	8.9
5	91.0	91.7	17.7	58.6	64.7	34.9	35.4	47.3	23.9	17.2	30.9	13.3
15	83.8	84.8	20.4	54.3	60.8	30.4	23.8	27.6	9.7	7.0	17.0	10.2
30	92.2	85.0	21.6	60.5	64.8	31.9	9.8	8.4	8.0	2.2	7.1	3.3
60	78.4	73.4	20.4	55.4	56.9	26.3	4.4	4.0	6.8	1.1	4.1	2.3
90	86.9	78.7			82.8	5.8	1.2	0.7			0.9	0.4
120	59.8	86.4	18.9	64.3	57.3	28.1	1.7	0.7	9.4	0.0	2.9	4.4
180	77.7	61.5	20.5	71.0	57.7	25.7	1.2	0.0	5.3	0.0	1.6	2.5
240	92.4	106.7	19.3	55.6	68.5	39.2	2.1	0.0	7.7	0.0	2.4	3.6
300	74.5	98.6	21.7	56.4	62.8	32.4	0.0	2.4	7.6	0.0	2.5	3.6

Procalcitonin	Concentration (ng/mL)						Concentration (ng/mL)					
Time (min)	Control 1	Control 2	Control 3	Control 4	Average Control	SD Control	CytoSorb 1	CytoSorb 2	CytoSorb 3	CytoSorb 4	Average CytoSorb	SD CytoSorb
0	0.465	0.507	9.73454	5.153	3.964885	4.431204	8.025	8.6685	10.26284	3.838	7.698585	2.740243
5	0.529	0.511	13.81888	4.448	4.82672	6.274239	5.7615	3.7995	7.17956	1.986	4.68164	2.269387
15	0.539	0.57	12.12204	3.934	4.29126	5.458204	3.0315	1.7805	7.16742	1.211	3.297605	2.689598
30	0.426	0.562	10.26284	4.413	3.91596	4.617315	1.4505	1.1985	7.13064	0.725	2.62616	3.018009
60	0.418	0.476	12.41464	4.514	4.45566	5.641783	0.7455	0.783	5.35394	0.445	1.83186	2.352921
90	0.466			4.585	2.5255	2.912573	0.654	0.5535		0.411	0.5395	0.122103
120	0.466	0.583	11.18368	4.673	4.22642	5.033825	0.6045	0.426	0.57774	0.231	0.45981	0.1716
180	0.391	0.593	13.41188	4.778	4.79347	6.091057	0.426	0.345	0.04508	0.148	0.24102	0.17519
240	0.504	0.642	10.46952	5.258	4.21838	4.716802	0.333	0.345	0	0.042	0.18	0.184461
300	0.504	0.479	11.92494	5.211	4.529735	5.408887	0.333	0.2745	0	0.156	0.190875	0.147017

SPE-B	Concentration (ng/mL)						Concentration (ng/mL)					
Time (min)	Control 1	Control 2	Control 3	Control 4	Average Control	SD Control	CytoSorb 1	CytoSorb 2	CytoSorb 3	CytoSorb 4	Average CytoSorb	SD CytoSorb
0	105.6	119.6	148.0	97.0	117.5	22.3	156.3	166.8	138.0	258.0	179.8	53.5
5	82.9	68.0	73.0	54.0	69.5	12.0	98.6	93.9	36.0	211.0	109.9	73.2
15	93.6	109.3	144.0	209.0	139.0	51.2	69.4	83.7	32.0	61.0	61.5	21.8
30	92.0	109.9	32.0	305.0	134.7	118.3	68.5	67.5	0.0	0.0	34.0	39.2
60	118.6	104.7	91.0	150.0	116.1	25.3	59.7	73.2	0.0	0.0	33.2	38.8
90	108.1	108.4			108.2	0.2	44.5	32.3	0.0	0.0	19.2	22.7
120	117.3	115.4	79.0	101.0	103.2	17.7	54.3	27.6	0.0	0.0	20.5	26.0
180	113.8	89.0	279.0	116.0	149.4	87.2	41.1	16.9	0.0	0.0	14.5	19.4
240	104.9	99.1	169.0	305.0	169.5	95.7	31.1	19.2	0.0	0.0	12.6	15.3
300	119.5	78.2	177.0	93.0	116.9	43.5	25.5	16.2	0.0	0.0	10.4	12.6

TSST-1	Concentrat			Control			Concentration (ng/mL)					
Time (min)	Control 1	Control 2	Control 3	Control 4	Average Control	SD Control	CytoSorb 1	CytoSorb 2	CytoSorb 3	CytoSorb 4	Average CytoSorb	SD CytoSorb
0	1964	2215	783	783	1436.3	761.2	2230.0	2234.0	698.0	672.0	1458.5	893.2

5	2226	2100.5	536	680	1385.6	901.3	1898.0	1746.5	429.0	523.0	1149.1	780.7
15	omit	omit	465	635	550.0	120.2	1660.5	1448.5	369.0	416.0	973.5	676.7
30	2209	2132	726	637	1426.0	861.0	1345.0	1225.0	201.0	446.0	804.3	566.2
60	2316.5	2215.5	706	483	1430.3	970.2	795.0	806.0	65.0	147.0	453.3	402.4
90	2190	2437			2313.5	174.7	602.5	635.5			619.0	23.3
120	2415.5	2564	584	177	1435.1	1230.6	452.5	422.0	37.0	111.0	255.6	212.3
180	2335	2505.5	884	1521	1811.4	752.8	198.5	257.5	0.0	0.0	114.0	133.8
240	2347.5	2322.5	661	1465	1699.0	804.5	89.0	302.5	0.0	0.0	97.9	142.7
300	2322.5	2436.5	631	568	1489.5	1029.1	0.0	143.0	0.0	0.0	35.8	71.5

HMGB-1	**ntration (ng/mL)**						**Concentration (ng/mL)**					**Perc**
**Time (min)**	**Control 1**	**Control 2**	**Control 3**	**Control 4**	**Average Control**	**SD Control**	**CytoSorb 1**	**CytoSorb 2**	**CytoSorb 3**	**CytoSorb 4**	**Average CytoSorb**	**SD CytoSorb**
0	91.3	86.2	10.0	3.3	47.7	47.5	34.8	38.0	34.2	36.6	35.9	1.8
5	96.3	74.0	10.6	3.3	46.0	46.2	31.3	33.8	27.7	33.1	31.5	2.7
15	95.9	85.7	5.3	3.3	47.5	50.1	27.8	33.6	25.3	13.3	25.0	8.5
30	87.0	82.5	6.4	2.9	44.7	46.3	23.8	29.8	23.3	15.5	23.1	5.9
60	94.1	84.9	7.8	3.8	47.6	48.5	22.3	37.7	15.5	11.6	21.8	11.5
90	99.0	93.1			96.0	4.1	17.6	25.7	14.1	11.9	17.3	6.0
120	98.4	87.1	16.4	2.9	51.2	48.5	15.5	14.0	14.5	12.5	14.1	1.2
180	92.4	83.1	7.7	3.3	46.6	47.7	13.8	10.8	7.1	9.5	10.3	2.8
240	87.0	64.0	8.4	3.5	40.7	41.3	10.8	6.0	8.3	5.4	7.6	2.4
300	89.5	75.3	11.3	3.4	44.9	43.8	8.9	3.2	4.5	6.9	5.9	2.5

Vitamin B12

Percent Remaining						Percent Remaining					
Control 1	Control 2	Control 3	Control 4	Average Control	SD Control	CytoSorb 1	CytoSorb 2	CytoSorb 3	CytoSorb 4	Average CytoSorb	SD CytoSorb
100.0	100.0	100.0	100.0	100.0	0.0	100.0	100.0	100.0	100.0	100.0	0.0
100.8	104.2	100.6	103.5	102.3	1.9	29.5	15.3	30.8	19.9	23.9	7.5
98.4	102.1	102.9	97.6	100.3	2.6	16.8	14.6	17.8	21.9	17.8	3.0
97.3	97.1	98.8	97.6	97.7	0.8	7.7	8.6	9.3	17.6	10.8	4.6
100.0	100.0	107.6	101.2	102.2	3.7	3.7	1.5	2.8	10.8	4.7	4.1
102.3	107.6	108.8	109.4	107.0	3.2	2.4	0.0	7.0	8.3	4.4	3.9
105.0	93.7	112.4	109.4	105.1	8.2	3.9	0.0	7.0	5.7	4.2	3.1
105.4	100.8	114.7	115.3	109.1	7.1	5.6	0.0	10.7	3.2	4.9	4.5

Aflatoxin B1

Percent Remaining						Percent Remaining					
Control 1	Control 2	Control 3	Control 4	Average Cc	SD Control	CytoSorb 1	CytoSorb 2	CytoSorb 3	CytoSorb 4	Average CytoSorb	SD CytoSorb
100	100	100	100	100	0	100	100	100	100	100	0
92.2	119.3	115.3	93.2	105.0	14.3	24.5	13.6	43.5	60.1	35.4	20.6
156.2	100.7	94.4	105.6	114.2	28.4	13.0	9.0	20.3	19.9	15.5	5.5
81.8	98.8	104.4	107.4	98.1	11.4	4.3	4.1	10.6	9.9	7.2	3.5
77.0	102.4	86.3	100.6	91.6	12.1	3.4	1.7	5.3	5.7	4.0	1.8
156.2	95.7	80.0	80.2	103.0	36.2	1.2	1.2	3.9	4.8	2.8	1.9
156.2	90.5	91.9	86.4	106.3	33.4	1.3	0.9	3.9	3.7	2.5	1.6
156.2	94.3	81.3	88.9	105.2	34.5	1.2	0.8	3.8	4.1	2.5	1.7

T2 Toxin

Percent Remaining						Percent Remaining					
Control 1	Control 2	Control 3	Control 4	Average Control	SD Control	CytoSorb 1	CytoSorb 2	CytoSorb 3	CytoSorb 4	Average CytoSorb	SD CytoSorb
100.0	100.0	100.0	100.0	100.0	0.0	100.0	100.0	100.0	100.0	100.0	0.0
95.3	91.4	95.0	104.0	96.4	5.4	4.1	1.3	9.8	9.4	6.1	4.2
86.2	97.3	94.1	109.3	96.7	9.6	0.0	0.8	4.0	2.3	1.8	1.8
83.4	101.1	107.6	103.2	98.8	10.6	0.0	0.0	1.3	0.7	0.5	0.6

88.8	87.7	94.1	100.8	92.8	6.0	0.0	0.0	0.5	0.2	0.2	0.2
77.3	96.3	95.0	106.6	93.8	12.2	0.0	0.0	0.1	0.0	0.0	0.1

IL-6

Percent Remaining						Percent Remaining					
**Control 1**	**Control 2**	**Control 3**	**Control 4**	**Average Co**	**SD Control**	**CytoSorb 1**	**CytoSorb 2**	**CytoSorb 3**	**CytoSorb 4**	**Average Cy**	**SD CytoSorb**
100	100	100	100	100	0	100	100	100	100	100	0
104	93	100	97	99	5	74	64	86	43	67	18
106	101	99	89	99	7	62	58	71	37	57	14
108	101	97	92	100	7	50	51	62	26	47	15
108	102	103	87	100	9	39	37	48	19	36	12
114	107	104	87	103	12	25	21	30	11	22	8
110	110	112	91	106	10	18	14	21	7	15	6
118	108	104	97	107	9	12	11	16	6	11	4
117	120	106	95	109	11	11	9	12	5	9	3

MIP-1α

Percent Remaining						Percent Remaining					
**Control 1**	**Control 2**	**Control 3**	**Control 4**	**Average Control**	**SD Control**	**CytoSorb 1**	**CytoSorb 2**	**CytoSorb 3**	**CytoSorb 4**	**Average CytoSorb**	**SD CytoSorb**
100.0	100.0	100.0	100.0	100.0	0.0	100.0	100.0	100.0	100.0	100.0	0.0
97.3	111.8	92.9	83.8	96.5	11.7	65.7	60.8	64.4	70.1	63.6	2.5
109.8	112.4	88.8	77.2	97.0	16.9	36.6	40.0	37.0	67.2	37.9	1.9
93.0	116.7	91.7	73.8	93.8	17.6	15.8	19.2	19.6	70.1	18.2	2.1
89.8	115.6	87.5	82.1	93.8	14.9	4.5	11.5	6.2	38.7	7.4	3.7
89.8	111.8	83.8	81.1	91.6	13.9	0.0	7.7	0.0	31.4	2.6	4.4
98.0	109.1	89.2	77.0	93.3	13.6	0.0	6.9	0.0	21.9	2.3	4.0
109.0	114.5	89.2	77.4	97.5	17.3	0.0	6.2	0.0	16.8	2.1	3.6
108.6	115.1	90.8	75.6	97.5	17.8	0.0	6.9	0.0	15.3	2.3	4.0

IFN-ɣ

Percent Remaining						Percent Remaining					
**Control 1**	**Control 2**	**Control 3**	**Control 4**	**Average Control**	**SD Control**	**CytoSorb 1**	**CytoSorb 2**	**CytoSorb 3**	**CytoSorb 4**	**Average CytoSorb**	**SD CytoSorb**
100.0	100.0	100.0	100.0	100.0	0.0	100.00	100.00	100.00	100.00	100.00	0.00
86.5	91.9	91.6	92.1	90.5	2.7	68.75	91.85	87.50	75.00	80.78	10.74

89.9	86.1	93.7	93.1	90.7	3.5	64.42	81.24	84.36	70.19	75.05	9.34
84.6	89.3	91.6	88.1	88.4	2.9	53.85	71.13	80.87	53.85	64.92	13.39
84.0	88.7	87.4	86.1	86.6	2.0	44.23	58.01	80.98	46.15	57.34	16.90
75.5	76.9	87.4	86.1	81.5	6.1	19.71	36.06	70.87	29.81	39.11	22.22
77.5	69.9	80.0	85.1	78.1	6.3	13.46	25.20	58.49	20.19	29.34	20.02
75.5	59.7	82.1	84.2	75.4	11.1	9.13	13.78	51.49	13.46	21.97	19.80
75.5	50.7	84.2	85.1	73.9	16.1	9.13	10.83	44.58	7.69	18.06	17.73

TNF-α

Percent Remaining						Percent Remaining					
Control 1	Control 2	Control 3	Control 4	Average Control	SD Control	CytoSorb 1	CytoSorb 2	CytoSorb 3	CytoSorb 4	Average	SD
100.0	100.0	100.0	100.0	100.0	0.0	100.0	100.0	100.0	100.0	100.0	0.0
94.3	96.1	95.2	103.1	97.2	4.0	89.8	93.4	92.1	90.5	91.5	1.6
96.5	121.1	92.2	101.5	102.8	12.8	85.0	95.2	85.7	90.8	89.2	4.8
99.2	112.9	95.3	104.6	103.0	7.6	79.3	87.5	75.3	82.2	81.1	5.1
100.5	116.1	98.2	107.5	105.5	8.0	75.8	86.0	78.1	78.0	79.5	4.5
93.4	87.5	101.3	110.5	98.2	10.0	69.1	86.2	68.1	89.7	78.3	11.3
89.0	91.8	105.7	108.2	98.7	9.7	64.3	80.8	65.9	66.6	69.4	7.7
98.6	108.6	102.2	109.8	104.8	5.3	61.0	74.2	56.6	62.4	63.6	7.5
96.6	81.8	97.8	102.8	94.7	9.1	65.4	68.8	48.2	60.2	60.7	9.0

α-toxin

Percent Remaining						Percent Remaining					
Control 1	Control 2	Control 3	Control 4	Average Control	SD Control	CytoSorb 1	CytoSorb 2	CytoSorb 3	CytoSorb 4	Average CytoSorb	SD CytoSorb
100.0	100.0	100.0	100.0	100.0	0.0	100.0	100.0	100.0	100.0	100.0	0.0
106.3	89.5	65.4	92.4	88.4	17.0	82.0	91.7	85.4	82.2	85.3	4.5
108.3	76.1	67.6	85.2	84.3	17.5	75.4	95.0	78.9	83.4	83.2	8.5
101.3	97.6	56.7	89.7	86.3	20.3	50.7	89.8	56.4	60.7	64.4	17.4
78.1	129.4	63.6	104.2	93.8	29.1	45.3	63.8	38.0	41.2	47.1	11.5
80.3	103.5	59.9	113.1	89.2	23.9	55.7	29.4	32.9	42.6	40.2	11.8
90.6	81.0	67.9	105.1	86.1	15.7	47.1	20.6	25.0	40.5	33.3	12.5
105.3	86.3	78.8	98.3	92.2	11.9	30.8	9.7	24.5	23.4	22.1	8.9
112.5	75.2	69.4	103.1	90.0	21.0	15.4	13.3	29.6	17.1	18.9	7.4

90.3	69.6	62.6	90.1	78.2	14.2	15.4	20.5	18.7	12.2	16.7	3.7

S100-A8

Percent Remaining						Percent Remaining					
Control 1	Control 2	Control 3	Control 4	Average Control	SD Control	CytoSorb 1	CytoSorb 2	CytoSorb 3	CytoSorb 4	Average CytoSorb	SD CytoSorb
100.0	100.0	100.0	100.0	100.0	0.0	100.0	100.0	100.0	100.0	100.0	0.0
102.2	92.4	112.6	90.0	99.3	10.3	81.6	81.9	70.5	63.6	74.4	8.9
101.8	79.2	111.7	75.8	92.1	17.4	61.4	70.1	70.5	60.0	65.5	5.6
97.7	72.4	99.6	65.8	83.9	17.3	40.5	49.6	38.7	42.0	42.7	4.8
97.4	89.1	62.7	79.5	82.2	14.9	30.3	32.8	25.0	24.8	28.2	4.0
88.9	76.1		70.2	78.4	9.5	17.7	22.6			20.1	3.5
97.5	85.4	68.2	66.4	79.4	14.8	15.0	16.0	8.1	23.1	15.6	6.2
86.2	85.8	52.5	64.2	72.2	16.7	10.2	12.0	3.2	21.8	11.8	7.7
87.2	82.9	54.7	73.1	74.5	14.5	7.5	8.9	4.3	22.6	10.8	8.1
85.2	78.4	66.3	71.8	75.4	8.1	6.0	6.8	6.4	17.2	9.1	5.4

C5a

Percent Remaining						Percent Remaining					
Control 1	Control 2	Control 3	Control 4	Average Control	SD Control	CytoSorb 1	CytoSorb 2	CytoSorb 3	CytoSorb 4	Average CytoSorb	SD CytoSorb
100.0	100.0	100.0	100.0	100.0	0.0	100.0	100.0	100.0	100.0	100.0	0.0
106.4	131.6	111.6	96.4	111.5	14.8	50.5	73.8	46.4	32.4	50.8	17.2
98.0	121.8	128.8	89.4	109.5	18.8	34.0	43.0	18.8	13.1	27.2	13.7
107.9	122.0	135.9	99.6	116.3	16.0	14.0	13.0	15.5	4.2	11.7	5.1
91.8	105.3	128.4	91.2	104.2	17.4	6.3	6.3	13.2	2.1	7.0	4.6
101.7	113.0			107.3	8.0	1.7	1.0			1.4	0.5
69.9	124.0	119.1	105.8	104.7	24.4	2.4	1.0	18.3	0.0	5.4	8.6
91.0	88.3	129.3	116.9	106.4	20.0	1.7	0.0	10.3	0.0	3.0	4.9
108.1	153.1	121.3	91.6	118.5	26.0	2.9	0.0	14.9	0.0	4.5	7.1
87.2	141.5	137.0	92.8	114.6	28.6	0.0	3.7	14.7	0.0	4.6	6.9

Procalcitonin

		Percent Remaining						Percent Remaining			
Control 1	Control 2	Control 3	Control 4	Average Control	SD Control	CytoSorb 1	CytoSorb 2	CytoSorb 3	CytoSorb 4	Average CytoSorb	SD CytoSorb
100	100	100	100	100	0	100	100	100	100	100	0
113.7634	100.789	141.9572	86.31865	110.7071	23.65781	71.79439	43.83111	69.95685	51.7457	59.33202	13.73594
115.914	112.426	124.5261	76.34388	107.3025	21.25634	37.7757	20.53989	69.83856	31.55289	39.92676	21.17637
91.6129	110.8481	105.4271	85.63943	98.38188	11.73642	18.07477	13.82592	69.48018	18.89005	30.06773	26.3686
89.89247	93.8856	127.5319	87.59946	99.72735	18.71744	9.28972	9.032705	52.16821	11.59458	20.5213	21.12936
100.2151			88.97729	94.59617	7.946296	8.149533	6.385188	0	10.7087	6.310856	4.566334
100.2151	114.9901	114.8866	90.68504	105.1942	11.90533	7.53271	4.914345	5.629436	6.01876	6.023813	1.105026
84.08602	116.9625	137.7762	92.72269	107.8869	24.30482	5.308411	3.979927	0.439255	3.856175	3.395942	2.077851
108.3871	126.6272	107.5502	102.0376	111.1505	10.69535	4.149533	3.979927	0	1.09432	2.305945	2.080581
108.3871	94.47732	122.5013	101.1256	106.6228	12.0135	4.149533	3.166638	0	4.064617	2.845197	1.948225

SPE-B

		Percent Remaining						Percent Remaining			
Control 1	Control 2	Control 3	Control 4	Average Control	SD Control	CytoSorb 1	CytoSorb 2	CytoSorb 3	CytoSorb 4	Average CytoSorb	SD CytoSorb
100.0	100.0	100.0	100.0	100.0	0.0	100.0	100.0	100.0	100.0	100.0	0.0
78.5	56.9	49.3	55.7	60.1	12.7	63.1	56.3	26.1	81.8	56.8	23.1
88.6	91.4	97.3	215.5	123.2	61.6	44.4	50.2	23.2	23.6	35.4	14.0
87.1	91.9	21.6	314.4	128.8	127.9	43.8	40.4	0.0	0.0	21.1	24.4
112.3	87.5	61.5	154.6	104.0	39.6	38.2	43.9	0.0	0.0	20.5	23.8
102.3	90.6			96.5	8.3	28.4	19.4	0.0	0.0	12.0	14.3
111.0	96.5	53.4	104.1	91.3	25.9	34.7	16.5	0.0	0.0	12.8	16.6
107.8	74.4	188.5	119.6	122.6	47.9	26.3	10.1	0.0	0.0	9.1	12.4
99.3	82.9	114.2	314.4	152.7	108.6	19.9	11.5	0.0	0.0	7.8	9.7
113.1	65.4	119.6	95.9	98.5	24.2	16.3	9.7	0.0	0.0	6.5	8.0

TSST-1

		Percent Remaining						Percent Remaining			
Control 1	Control 2	Control 3	Control 4	Average Control	SD Control	CytoSorb 1	CytoSorb 2	CytoSorb 3	CytoSorb 4	Average CytoSorb	SD CytoSorb
100.0	100.0	100.0	100.0	100.0	0.0	100.0	100.0	100.0	100.0	100.0	0.0

113.3	94.8	68.5	86.8	90.9	18.6	85.1	78.2	61.5	77.8	75.6	10.0
		59.4	81.1	70.2	15.4	74.5	64.8	52.9	61.9	63.5	8.9
112.5	96.3	92.7	81.4	95.7	12.9	60.3	54.8	28.8	66.4	52.6	16.5
117.9	100.0	90.2	61.7	92.5	23.5	35.7	36.1	9.3	21.9	25.7	12.8
111.5	110.0			110.8	1.0	27.0	28.4			27.7	1.0
123.0	115.8	74.6	22.6	84.0	46.1	20.3	18.9	5.3	16.5	15.3	6.8
118.9	113.1	112.9	194.3	134.8	39.7	8.9	11.5	0.0	0.0	5.1	6.0
119.5	104.9	84.4	187.1	124.0	44.5	4.0	13.5	0.0	0.0	4.4	6.4
118.3	110.0	80.6	72.5	95.3	22.2	0.0	6.4	0.0	0.0	1.6	3.2

HMGB-1

**cent Remaining**						**Percent Remaining**					
**Control 1**	**Control 2**	**Control 3**	**Control 4**	**Average Control**	**SD Control**	**CytoSorb 1**	**CytoSorb 2**	**CytoSorb 3**	**CytoSorb 4**	**Average CytoSorb**	**SD CytoSorb**
100.0	100.0	100.0	100.0	100.0	0.0	100.0	100.0	100.0	100.0	100.0	0.0
105.4	85.8	105.8	98.7	98.9	9.4	90.0	88.8	81.0	90.6	87.6	4.5
105.0	99.4	52.7	101.3	89.6	24.7	80.1	88.3	74.1	36.4	69.7	22.9
95.2	95.7	64.3	87.4	85.6	14.7	68.4	78.3	68.2	42.3	64.3	15.4
103.0	98.5	77.6	113.9	98.2	15.2	64.1	99.1	45.4	31.6	60.1	29.2
108.3	108.0			108.2	0.3	50.6	67.6	41.3	32.6	48.0	15.0
107.7	101.0	163.9	88.7	115.3	33.4	44.4	36.9	42.4	34.1	39.5	4.8
101.2	96.4	76.7	98.7	93.3	11.2	39.6	28.4	20.9	26.1	28.7	7.9
95.3	74.2	83.4	106.3	89.8	14.0	31.0	15.7	24.3	14.9	21.5	7.6
98.0	87.3	112.4	103.8	100.4	10.5	25.7	8.4	13.2	18.7	16.5	7.4
